# The P2X7 receptor and pannexin-1 are involved in glucose-induced autocrine regulation in β-cells

**DOI:** 10.1038/s41598-018-27281-9

**Published:** 2018-06-12

**Authors:** Marco Tozzi, Anna T. Larsen, Sofie C. Lange, Andrea Giannuzzo, Martin N. Andersen, Ivana Novak

**Affiliations:** Section for Cell Biology and Physiology, August Krogh Building, Department of Biology, University of Copenhagen, Copenhagen, Denmark

## Abstract

Extracellular ATP is an important short-range signaling molecule that promotes various physiological responses virtually in all cell types, including pancreatic β-cells. It is well documented that pancreatic β-cells release ATP through exocytosis of insulin granules upon glucose stimulation. We hypothesized that glucose might stimulate ATP release through other non-vesicular mechanisms. Several purinergic receptors are found in β-cells and there is increasing evidence that purinergic signaling regulates β-cell functions and survival. One of the receptors that may be relevant is the P2X7 receptor, but its detailed role in β-cell physiology is unclear. In this study we investigated roles of the P2X7 receptor and pannexin-1 in ATP release, intracellular ATP, Ca^2+^ signals, insulin release and cell proliferation/survival in β-cells. Results show that glucose induces rapid release of ATP and significant fraction of release involves the P2X7 receptor and pannexin-1, both expressed in INS-1E cells, rat and mouse β-cells. Furthermore, we provide pharmacological evidence that extracellular ATP, via P2X7 receptor, stimulates Ca^2+^ transients and cell proliferation in INS-1E cells and insulin secretion in INS-1E cells and rat islets. These data indicate that the P2X7 receptor and pannexin-1 have important functions in β-cell physiology, and should be considered in understanding and treatment of diabetes.

## Introduction

Pancreatic β-cells are the only source of insulin in the body, and thus they have a key role in whole body metabolic homeostasis. Regulation of insulin secretion is complex; intracellular ATP has a central role, but there is now solid evidence that also extracellular ATP is an important regulator of β-cell functions. For example, extracellular nucleotides/sides can evoke insulin secretion, also independently of glucose, and this response is preserved in type-2 diabetes models^1^. There are two potential sources of extracellular ATP for stimulating β-cells: ATP co-releases with transmitters from nerve terminals, and ATP released from insulin-containing granules^2–5^. In particular, the latter process is well investigated and it has been shown that ATP is stored in vesicles and upon release can reach local concentrations in micromolar range^2–5^. However, it appears that release of small molecules like ATP (and GABA) precedes release of peptide cargo and acts with positive feedback/autocrine stimulation^6,7^. Accumulation of ATP into vesicles is thought to occur via vesicular nucleotide transporter, VNUT/SLC17A9, and knockdown of VNUT leads to diminished glucose-responsive ATP release, though described effects on insulin release are disparate^8,9^. Moreover, it cannot be excluded that β-cells can also release ATP by other mechanisms, which can include connexins, pannexin-1, maxi-anion channels, cell volume and mechanosensitive pathways^10,11^. In particular, several recent studies focus on pannexin-1 as a major ATP efflux pathway^12,13^. Thorough investigations of such alternative ATP-release pathways in β-cells are pending until now.

The pancreatic β-cells express a number of purinergic P2 (and adenosine) receptors that have different effects on cell functions. In rodent β-cells and pancreas the P2Y1 and P2Y6 receptors stimulate insulin secretion^14^, while the mouse P2Y13 receptor inhibits secretion^15^ and also causes glucolipotoxicity^16,17^. In human β-cells, recent studies indicate that the P2X3 receptor regulates insulin secretion in an autocrine fashion^18^, though the P2Y1 receptor as a key receptor in autocrine regulation of mouse and human cells has been revived^19,20^. Regarding regulation of β-cells mass, the number of studies are not yet extensive but proliferative, cytoprotective and apoptotic function of some receptors, for example, P2Y6 and P2Y13 receptors, have been described^17,21,22^.

One interesting and potentially important receptor is the P2X7 receptor (P2X7R) because it plays a central role in both health and a wide spectrum of disorders, such as central nervous system diseases, pain, osteoporosis, cancer and inflammation^23–27^. The receptor is highly polymorphic and recent studies show that several single nucleotide polymorphisms (SNPs) in the receptor are associated with osteoporosis, multiple myeloma, leukemia, pain and bipolar diseases^28–32^. The P2X7R has different modes of operation (cation-selective channels *vs*. large lytic pores); and it plays a dual role in cell survival (proliferation *vs*. cell death) as initially described by Di Virgilio’s group on lymphocytes/lymphoid cells^33,34^ and confirmed by many others on a variety of cells^35–40^. Since the P2X7R is a key receptor in inflammation and it is expressed in immune cells^41^, it has been ascribed a role in inflammation and diabetes^1,25,27,42^. Based on studies of P2X7R expressing lymphocytes in non-obese diabetic (NOD) mice, it was proposed that the receptor is an important candidate in type-1 diabetes in these mice^43,44^. Further, a knockout of the P2X7R prevented streptozotocin-induced type-1 diabetes in mice^45^, and targeting the receptor in T-cells with the inhibitor, oxidized-ATP, delayed islet allograft rejection^46^.

Whether the P2X7R is expressed in β-cells and which function it may have is not entirely clear. Some studies report immunolocalization of the P2X7R in rodent α-cells and others in β-cells^47–49^. However, the P2X7R was detected on mRNA and protein level in the rat β-cell line INS-1E, but the potentiating and inhibitory effects on insulin secretion were ascribed to P2Y4 and P2X3 receptors, respectively^50^. A P2X7-like receptor was reported for the hamster cell line HIT-T15, where its stimulation induced cation fluxes, pore formation and apoptosis after 24 h of stimulation^51^. P2X7R knockout mice had lower β-cell mass, impaired glucose tolerance and defective insulin and IL-1β and IL-1Ra secretion^49^. The P2X7R was found by *in situ* hybridization in human islets, where ATP increased insulin secretion, while unspecific blockers BBG and KN-62 had insignificant effects on insulin secretion^18^. The authors favored the interpretation that the P2X3 receptor was the main autocrine signaling pathway. A recent study indicates that variations in glucose homeostasis traits are associated with P2X7R polymorphisms in humans and mice^52^. In particular, hypoactive SNP polymorphism (P451L) in mice leads to different glucose regulation under stress (glucose and insulin tolerance tests), which may reflect changes in inflammasome activation, release of cytokines and other indirect effects. Again, the role of P2X7R in immune system rather than in β-cells has been considered.

The aim of this study was to establish whether β-cells express functional P2X7 receptors and pannexin-1 (Panx1) and to determine whether these proteins play roles in ATP release, insulin secretion and cell survival. For this purpose we used INS-1E β-cell line, which is well established as a model for mechanistic studies. We find that glucose stimulated ATP release, which is sensitive to P2X7R and Panx1 inhibition, and that further autocrine signaling via the P2X7R affects calcium signaling, insulin secretion and β-cell proliferation.

## Results

### Expression of the P2X7 receptor and pannexin-1

**Figure 1:**
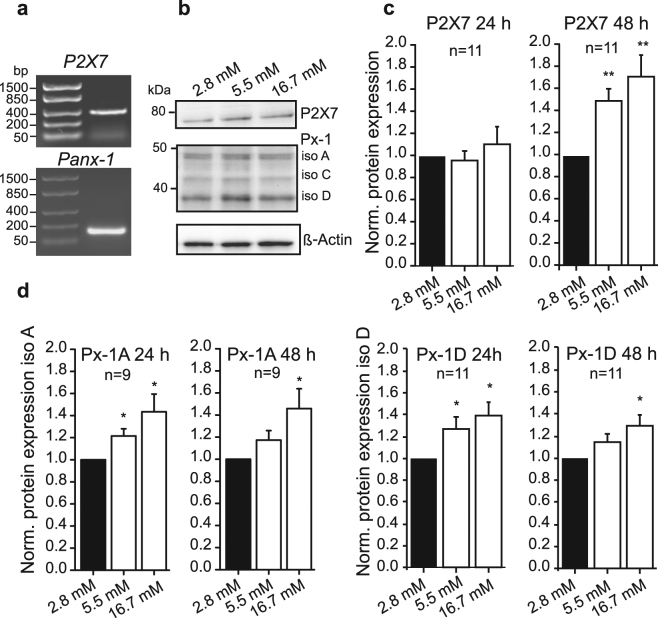
Expression of P2X7R and Panx1 in INS-1E cells. (**a**) Representative gel of *P2X7* and *Panx1* mRNA expression. (**b**) Representative Western blot of P2X7R and Panx1 expression in INS-1E cells grown in increasing glucose concentrations. Loading control was β-actin. The images (in a and b) are cropped from original full-length gels and blots that are shown in the supplementary material. (**c**,**d**) Western blot quantification of P2X7R and Panx1 (isoform A and D) expression in INS-1E cells grown in increasing glucose concentrations. The ratio of protein (P2X7R or Panx1) to β-actin were normalized to 2.8 mM glucose. Data represent means ± s.e.m. and n denotes the number of independent experiments and significant differences *p < 0.05 and **p < 0.01 are indicated.

Since P2X7R and Panx1 might be associated with ATP release, we first investigated whether these are expressed on mRNA and protein levels in INS-1E cells (Fig. 1). The PCR product shows that *P*2*X7* and *Panx1* are expressed in INS-1E cells (Fig. 1a). Protein expression of P2X7R and Panx1 in response to increasing glucose concentrations was determined using western blot and results are shown in Fig. 1b,c. The antibody against the intracellular C-terminal part of P2X7R recognized the full length isoform at about 75 kDa (Fig. 1b,c). Quantification of the full length P2X7R isoform shows that cells grown in high glucose (16.7 mM) for 48 h upregulate P2X7R expression compared to cells grown in low glucose (2.8 mM) (Fig. 1c). Anti-pannexin-1 antibody recognized three isoforms of the protein at around 48, 42 and 35 kDa, corresponding, respectively, to the three known rat isoforms (A, C and D)(Fig. 1b)^53^. Both Panx1A and D were strongly expressed and the expression of both isoforms was upregulated with increased glucose concentrations after 24 and 48 h (Fig. 1d).

**Figure 2:**
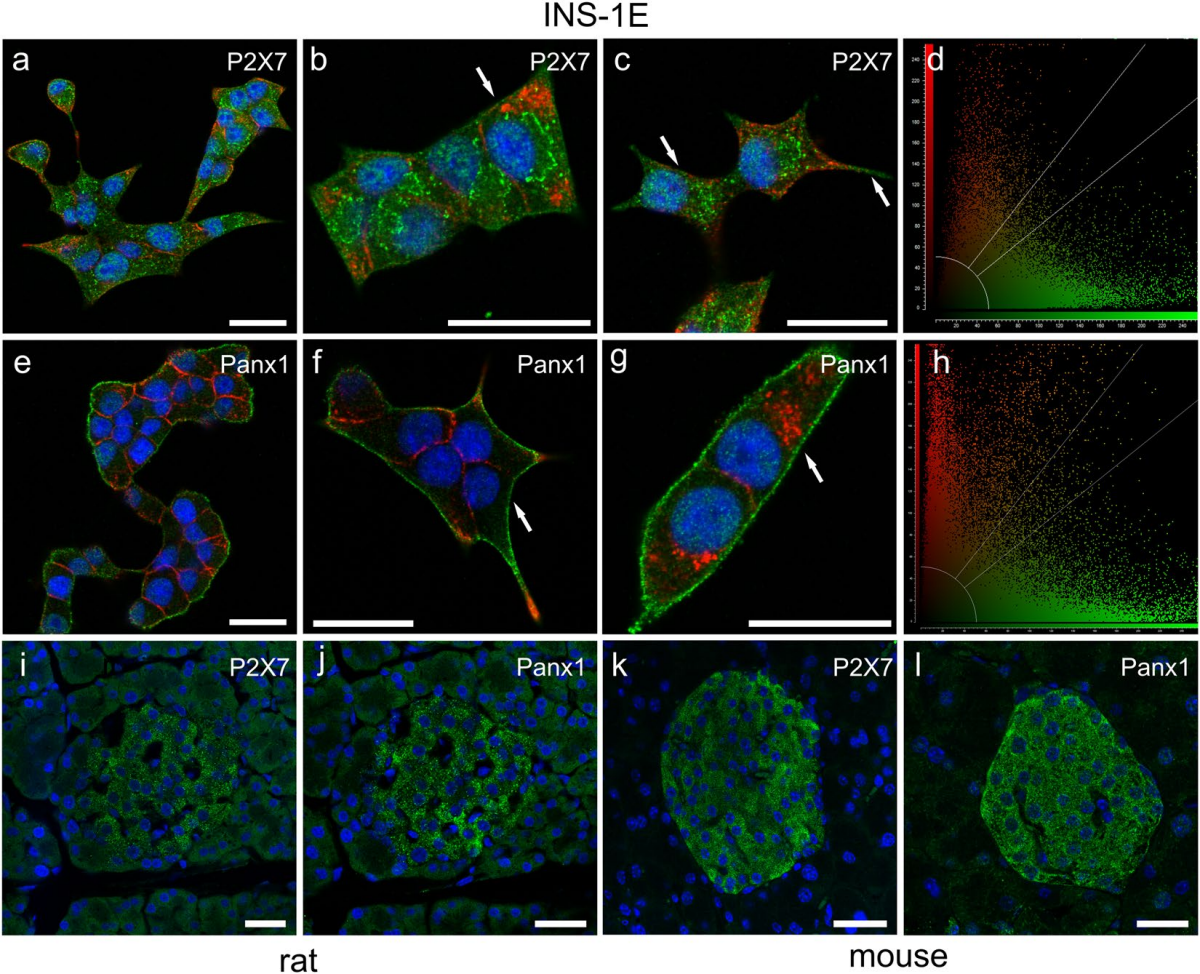
Immunolocalization of P2X7R and Panx1 in INS-1E cells, rat and mouse pancreas. Overlay images of INS-1E cells stained for P2X7R (**a–d**) and Panx1 (**e–h**) (green), insulin (red) and nuclei were stained with DAPI (blue). Co-localization graphs (**d**,**h**) show co-localization of P2X7R or Panx1 and insulin in corresponding images to the left (**c**,**g**). Person’s coefficient was x and y. Arrows indicate localization of the two proteins in the plasma membrane. Rat pancreas (**i**,**j**) and mouse pancreas (**k**,**l**) was stained for P2X7R (**i**,**k**) or Panx1 (**j**,**l**) (green) as above. All bars are 25 µm. Images shown here are representative of 3–10 images per slide on 3–9 independent staining experiments.

Localization of P2X7R and Panx1 in INS-1E cells as well as in native rat and mouse islets is shown by immunofluorescence in Fig. 2. The two proteins are expressed on all INS-1E cells as shown by representative images on cell colonies in Fig. 2a,e. The P2X7R is expressed weakly on plasma membranes in cluster-like formations (Fig. 2b,c arrows). These “clusters” may be located to lipid rafts, as is known to be the case for this receptor^54,55^. Furthermore, P2X7R is also found intracellulary in organelles most likely associated with receptor synthesis or endocytosis. We observed similar expression of recombinant rat P2X7R tagged with GFP in HEK293 cells^56^. On a whole-cell level analysis we found that there is weak co-localization of the P2X7R and insulin as shown in Fig. 2d (Pearson´s correlation coefficient of 0.13). In 16 images analyzed, the Pearson´s correlation coefficient was 0.24 ± 0.07. In contrast to P2X7R, Panx1 is strongly expressed on the plasma membrane (Fig. 2e,f) but some clusters are also present on the plasma membrane and intracellularly. The high-resolution image (Fig. 2g) and co-localization analysis (Fig. 2h, Pearson’s correlation coefficient of 0.10) indicate weak co-localization with insulin vesicles. In 13 images analyzed the coefficient was 0.20 ± 0.02. Figure 2i,j shows P2X7R and Panx1 immunoreactivity in rat islets, respectively, and Fig. 2k,l show the two proteins in mouse islets.

### Glucose stimulates fast ATP release that depends on the P2X7 receptor and pannexin-1

**Figure 3:**
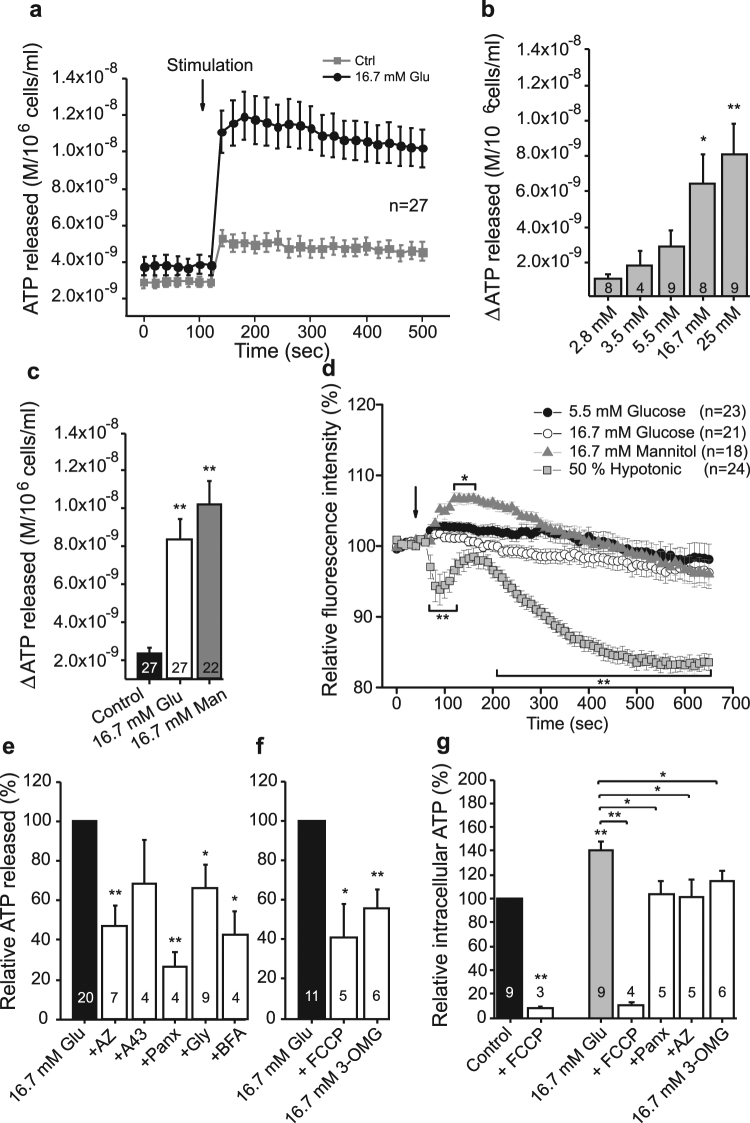
Effect of glucose, mannitol, metabolic and transport inhibitors on ATP release and intracellular ATP. (**a**) The time-course of ATP release after stimulation with 16.7 mM glucose and corresponding control (5.5 mM glucose). The arrow indicates stimulus application. (**b**) ATP released in response to increasing glucose concentrations shown as the difference between the basal extracellular ATP (in 2.8 mM glucose) and the peak release with the indicated glucose concentrations. (**c**) ATP release in response to high glucose (Glu) and mannitol (Man). The control glucose concentration was 5.5 mM and data show the difference in extracellular ATP before and after stimulation with the indicated factors. (**d**) Cell volume was measured as relative fluorescence intensity of calcein. The arrow indicates stimulation with 5.5 mM glucose, 16.7 mM glucose, 16.7 mM mannitol or hypotonic solutions. A decrease in fluorescence intensity indicates cell volume increases. Responses in each cell were normalized to the average of the baseline and corrected for bleaching. **(e,f)** The difference in extracellular ATP before and after stimulation with 16.7 mM glucose alone or in combination with a pre-treatment with the indicated inhibitors: AZ10606120 (10 μM), A438079 (10 μM), ^10^Panx (100 μM), glyoxylate (50 μM), bafilomycin A (1 μM), FCCP (5 μM) or 3-O-methylglucose. (**h**) Intracellular ATP was measured in cells incubated with 5.5 mM glucose (control) or 16.7 mM glucose with or without inhibitors as indicated. Data are normalized to the response with 16.7 mM glucose. All data are shown as mean values ± s.e.m. and significance is indicated *p < 0.05 and **p < 0.01.

Since pancreatic β-cells are exposed to various concentrations of blood glucose, we tested whether changes in glucose concentration affect ATP release in INS-1E cells. Figure 3a shows online live-cell measurement of ATP release. Note that addition of an equivalent volume of solution containing 5.5 mM glucose evoked a small ATP release, most likely due to mechanical disturbance caused by pipetting. Mechanical sensitivity of β-cells is shown in Supplementary Fig. 1. Nevertheless, an increase in the glucose concentration to 16.7 mM induced a rapid and large increase in the extracellular concentration of ATP (Fig. 3a). Furthermore, stimulating the cells with increasing glucose concentrations ranging from 2.8 mM glucose to 25 mM showed a dose-dependent increase in the ATP release (Fig. 3b).

We hypothesized that the rapid glucose effect on ATP release could be due to osmotic changes. Therefore, in another series of experiments we tested an equivalent concentration of mannitol and data are shown in Fig. 3c. Both 16.7 mM glucose and 16.7 mM mannitol induced ATP release. Next, we monitored cell volume changes with calcein. Figure 3d shows that glucose had no significant effect on cell volume, while mannitol caused transient cell volume decrease (fluorescence increase) followed by cell volume recovery. As a control for cell volume measurements, we exposed INS-1E cells to hypotonic solution. This caused cell swelling (fluorescence decrease), transient cell volume recovery, and then swelling/loss of fluorophore with prolonged hypotonic exposure. Therefore, although hyperosmotic stress (mannitol) could induce ATP release, ATP release due to equiosmolar glucose is not likely due to osmotic changes, as presumably glucose enters the cells and would be rapidly metabolized.

In the following series of experiments we studied ATP release mechanisms. As shown in Fig. 3e, glucose-stimulated ATP release was significantly diminished by the P2X7R inhibitor AZ10606120. Another inhibitor (A438079) also appeared to reduce ATP release, though the effect was not significant. The pannexin-1 mimetic inhibitory peptide ^10^Panx also significantly reduced glucose-stimulated ATP release (Fig. 3e). Glyoxylate, a putative inhibitor of vesicular nucleotide transporter (VNUT) and bafilomycin A, a V-ATPase inhibitor, both inhibited ATP release which indicates that also vesicular exocytosis could be involved in ATP release. However, none of the tested inhibitors had any significant effect on mannitol-stimulated ATP release (Supplementary Fig. 1). These results indicate that indeed glucose and mannitol stimulate ATP release through different mechanisms.

Furthermore, we investigated the role of glucose transport and metabolism on ATP release using the non-metabolizable glucose analogue 3-OMG and the mitochondrial uncoupler FCCP (Fig. 3f). Addition of FCCP or 16.7 mM 3-OMG induced significantly less ATP release compared to the corresponding concentration of glucose. We hypothesized that ATP release process correlates with intracellular ATP levels and therefore we measured intracellular ATP levels at the time-point when we detected peak ATP release. Indeed, as shown in Fig. 3g, 16.7 mM glucose increased intracellular ATP content compared to the control (5.5 mM glucose) and FCCP dramatically decreased it both in low and high glucose conditions. Addition of 16.7 mM 3-OMG did not increase intracellular ATP above the control level, thus indicating that glucose transport and metabolism was lower, and again correlated with lower ATP release. Lastly, inhibition of Panx1 and P2X7R significantly inhibited the high glucose-induced increase in intracellular ATP level, indicating that purinergic signaling was important in maintaining intracellular ATP as well as ATP release (Fig. 3e,g).

### P2X7R stimulates calcium responses and insulin secretion

**Figure 4:**
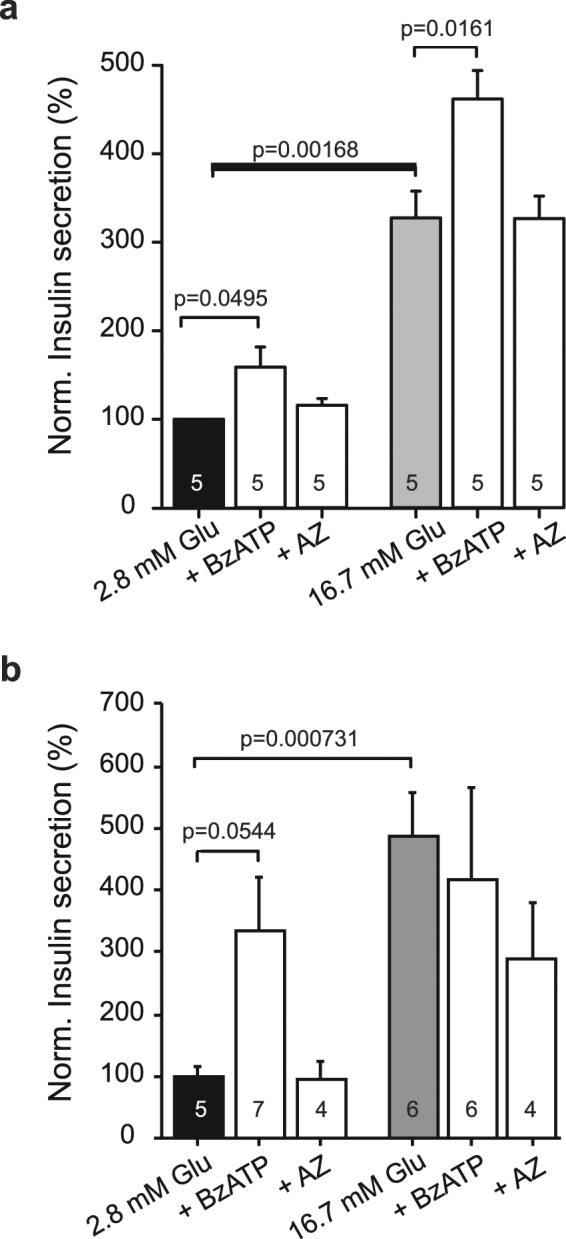
Role of P2X7R on insulin secretion in INS1-E cells and pancreatic islets. (**a**) Data show the effect of the P2X7R agonist BzATP (10 μM) or AZ10606120 (10 μM) on 30 min insulin secretion in INS1-E cells. Data are normalized to 2.8 mM glucose and show mean values ± s.e.m. of indicated number of independent experiments, and p-values are indicated. (**b**) Insulin secretion from pancreatic islets during 30 min stimulation with glucose, BzATP (10 μM) or AZ10606120 (10 μM). Data are normalized to 2.8 mM glucose and show mean values ± s.e.m. of the indicated number of experiments, and p-values are indicated.

To investigate the role of P2X7R in insulin secretion, we measured 30-min insulin secretion in INS-1E cells after P2X7R activation in low and high glucose concentrations. The P2X7R agonist BzATP (2′-3′-O-(4-benzoylbenzoyl)-ATP, 10 μM) significantly increased insulin secretion in both glucose concentrations (Fig. 4a). Perhaps surprisingly, the P2X7R inhibitor AZ10606120 had no significant effect on the insulin secretion in the two glucose concentrations.

We furthermore evaluated the role of the P2X7R in insulin secretion in isolated rat islets and Fig. 4b shows that at low glucose concentrations BzATP (10 μM) potentiated insulin secretion. Basal insulin secretion in 2.8 mM glucose was 0.93 ± 0.18 ng insulin/(islet*30 min) and insulin secretion in the presence of BzATP was 3.2 ± 1.1 ng insulin/(islet*30 min)(n = 5–7). High glucose concentrations (16.7 mM) increased insulin secretion to 5.7 ± 1.1 ng insulin/(islet*30 min) but BzATP had no further effect, most likely because β-cells were maximally stimulated.

**Figure 5:**
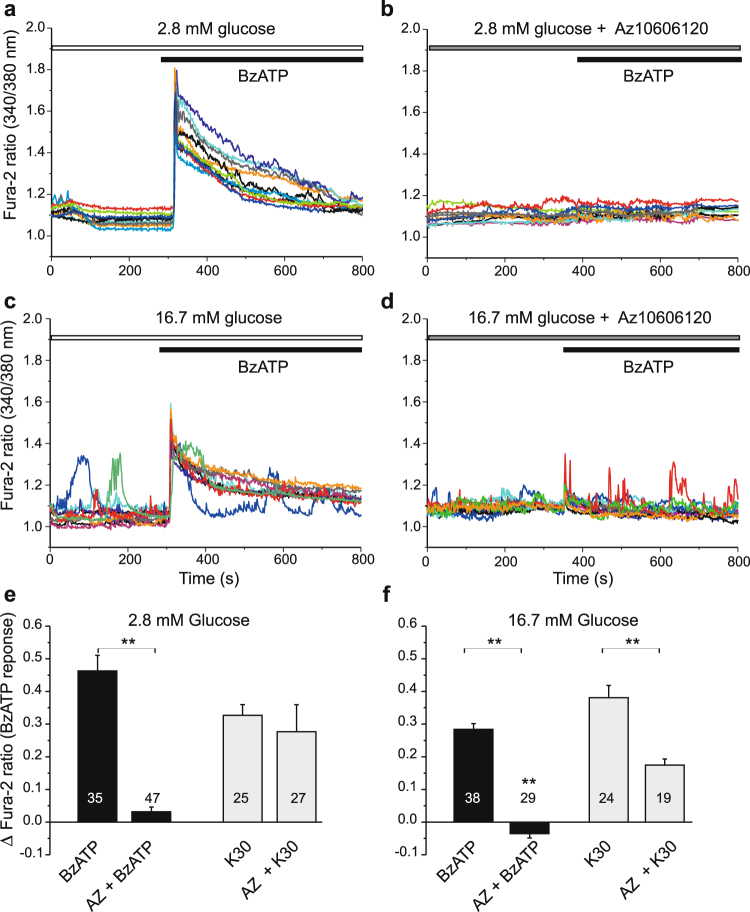
Effect of P2X7R and glucose on calcium transients. Representative recordings of intracellular Ca^2+^ (given as Fura-2 ratio) in INS-1E cells stimulated with BzATP (10 μM). (**a**) BzATP induced synchronous Ca^2+^ peaks in 2.8 mM glucose solutions. (**b**) This response was abolished by short pre-treatment with the P2X7R inhibitor (AZ10606120, 10 μM). (**c**) In high glucose (16.7 mM) many cells showed Ca^2+^ oscillations and BzATP induced synchronized Ca^2+^ peak. (**d**) Pre-treatment with AZ10606120 abolished the Ca^2+^ peak response. Each graph shows responses of 10 cells/experiment and each experiment was performed 3–4 times. (**e**,**f**) Bargraphs show the peak change means ± s.e.m. in fura-2 ratio in response to BzATP with/without pre-treatment with AZ10606120 (10 μM) for the two glucose concentrations indicated. The graph also shows the responses to a depolarizing K^+^ step (30 mM), which was carried out at the end of most experiments. Numbers indicate the number of cells analyzed in 3–4 independent experiments. Significant differences, **p < 0.01, are indicated.

It is well established that insulin secretion is activated by changes in intracellular Ca^2+^. Therefore, we investigated the effect of BzATP on intracellular Ca^2+^ signals in INS-1E cells loaded with Fura-2. Figure 5a shows that addition of 10 μM BzATP in 2.8 mM glucose buffer induced Ca^2+^ transients that were synchronized. Pre-treatment with the P2X7R inhibitor AZ10606120 almost abolished the synchronized Ca^2+^ transient (Fig. 5b,e), but Fura-2 signal indicated that intracellular Ca^2+^ was more unsteady (Fig. 5b). High glucose concentration (16.7 mM) induced characteristic Ca^2+^ oscillations in many cells, and addition of BzATP on top resulted in additional synchronized Ca^2+^ transients, which were abolished in cells pre-treated with the inhibitor (Fig. 5b,d). As a positive control at the end of the experiment, INS-1E cells were exposed to 30 mM K^+^, which is known to depolarize cell membrane voltage and increase Ca^2+^ influx via voltage-sensitive Ca^2+^ channels. The data showing effect of BzATP with/without AZ10606120 and K^+^ step are summarized in Fig. 5e,f.

**Figure 6:**
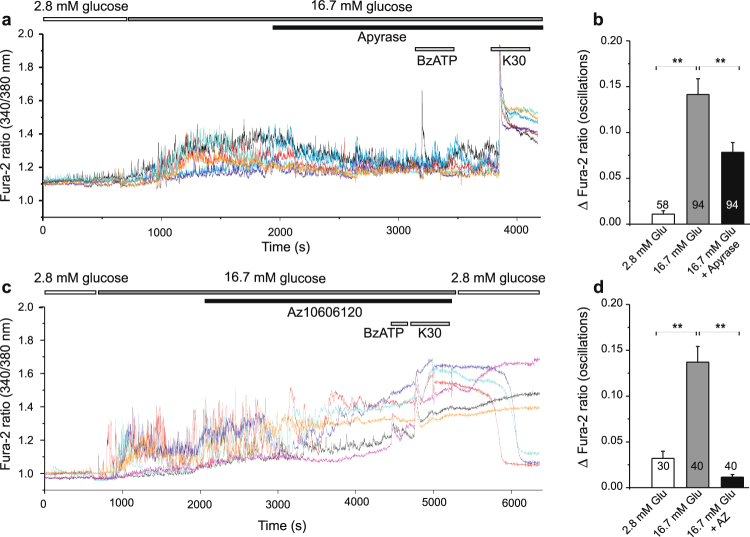
Effect of apyrase and AZ10606120 on glucose-induced calcium transients. (**a**) Increase in glucose (from 2.8 to 16.7 mM) induced calcium oscillations in many cells and these were reduced by introduction of apyrase (10 U/ml). BzATP (100 μM) had no, if any, effect while increase in K^+^ to 30 mM caused further increase in intracellular Ca^2+^. (**c**) Similar protocol as in (**a**) but instead of apyrase AZ10606120 (10 μM) was added to the bath. Oscillations in Ca^2+^ abated and Fura-2 ratio increased until medium was exchanged with fresh low glucose solution. (**b**, **d**) Bargraphs shows means ± s.e.m. of height of Ca^2+^ oscillations in 2.8 mM, 16.7 mM glucose with/without apyrase (**b**) or AZ10606120 (**d**). Numbers indicate the number of cells analyzed in 5 and 4 independent experiments, respectively. Significant differences, **p < 0.01, are indicated.

In additional series of experiments we tested whether extracellular ATP, released from cells in high-glucose conditions, could contribute to Ca^2+^ transients. Figure 6a,b shows that addition of apyrase to high-glucose solution was able to decrease the height of Ca^2+^ oscillations. Moreover, addition of BzATP had not much effect, most likely because it was hydrolyzed by apyrase. The K^+^ step though evoked expected Ca^2+^ increase. The P2X7R inhibitor, AZ10606120, had similar effect as apyrase on diminishing the Ca^2+^ oscillations induced by high-glucose (Fig. 6c,d). However, with prolonged exposure to AZ10606120 the Fura-2 ratio increased by about 0.3–0.6 (n = 4), indicating that the level of intracellular Ca^2+^ was rising. This was even more pronounced in cells that were pre-treated with AZ10606120 for about 30 min before Fura-2 imaging was started (data not shown). Furthermore, in the P2X7R inhibitor experiments, BzATP and K^+^ step had only small effects (Fig. 6c,d). Nevertheless, within experimental time frame INS-1E cells appeared healthy and Ca^2+^ signal recovered when cells were exposed to control 2.8 mM solution (Fig. 6c).

### P2X7R affects cell proliferation

**Figure 7:**
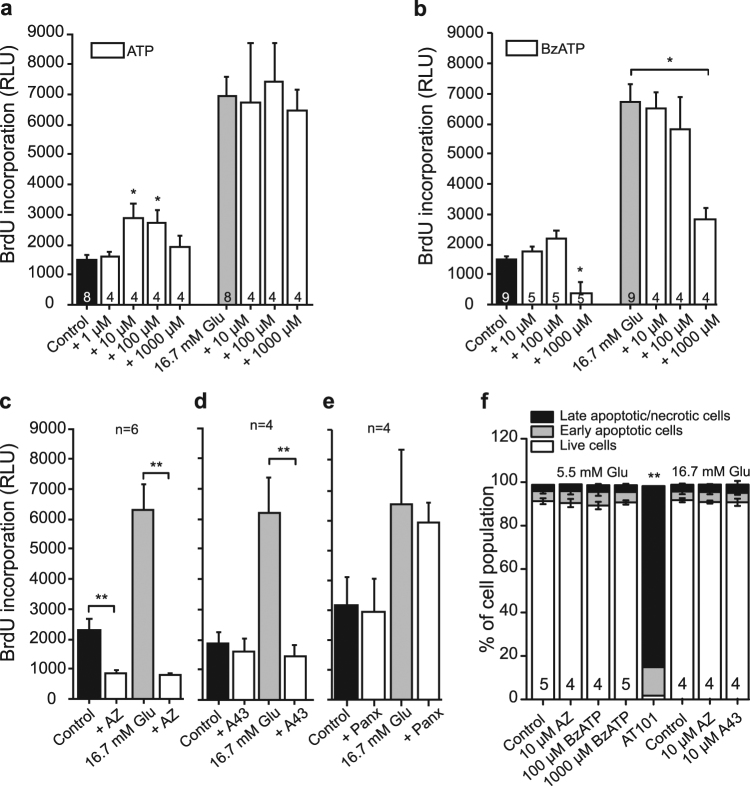
Effect of P2X7R on cell proliferation and viability. Data show cell proliferation measured by BrdU incorporation after 24 h. The black and grey bars show the effect of glucose alone and the white columns show the effect of increasing concentrations of ATP or BzATP. The control is 5.5 mM glucose. (**a,b**) Effect of exogenous ATP and the P2X7R agonist BzATP on cell proliferation. (**c–e**) Effect of the P2X7R inhibitors AZ10602120 (10 μM) and A438079 (10 μM), and pannexin-1 inhibitor ^10^Panx on cell proliferation at 5.5 mM and 16.7 mM glucose. (**f**) Cell viability was assayed by FACS analysis with Annexin V as apoptotic marker and propidium iodide as marker for necrotic or dead cells. Cells were incubated for 24 h with 5.5 mM or 16.7 mM of glucose in combination with the AZ10606120, A438079 or BzATP. AT101 (apoptotic inducer) was included as control. Representative FACS plots are shown in Supplementary Fig. 2. The bars show means ± s.e.m. of the percentage of cells in the indicated populations for 4–5 experiments. Late apoptotic and necrotic cells are shown as one population. Data represent means ± s.e.m. and n denotes the number of independent experiments and significant differences *p < 0.05 and **p < 0.01 are indicated.

In the following series of experiments we studied the effect of activation or inhibition of P2X7R on cell proliferation using BrdU assay. First, cells were incubated with increasing concentrations of the general P2 receptor agonist ATP in the presence of control glucose concentrations (5.5 mM) or in high glucose (16.7 mM). Figure 7a shows that high glucose increased BrdU incorporation and adding exogenous ATP had no further effect. However, 10 and 100 μM ATP significantly increased BrdU incorporation in control low glucose conditions. In order to explore whether this effect of ATP was due to P2X7R activation, BzATP, a more specific P2X7R agonist, was applied (Fig. 7b). There was a tendency that BzATP 10–100 μM also increased BrdU incorporation, but no significance was reached on this data set. Notably, increasing BzATP concentration to 1000 μM markedly reduced BrdU incorporation, both in control condition and when applied together with high glucose. These concentration-dependent and seemingly opposite effects of BzATP are one of the signatures of P2X7R multifunctionality (see discussion).

In order to verify whether cell proliferation involves P2X7R, we next tested the effect of the two P2X7R inhibitors AZ10606120 (10 μM) and A438079 (10 μM) (Fig. 7c,d). The application of AZ10606120 significantly decreased BrdU incorporation both in control and in high glucose conditions (Fig. 7c), and application of A437980 diminished the proliferative effect of high glucose (Fig. 7d). In order to confirm the general involvement of extracellular ATP on cell proliferation, we also tested the effect of a potent ATP-diphosphohydrolase (apyrase 10 U/ml) that hydrolyses ATP and ADP to AMP. As expected, apyrase inhibited BrdU incorporation in control and high glucose (Supplementary Fig. 1g). In contrast to P2X7R inhibitors, Panx1 inhibition seemed to have no effect on proliferation (Fig. 7e). Also mannitol was without significant effects on proliferation (Supplementary Fig. 1h).

Results in Fig. 7 indicate that high concentrations of BzATP (mM) and the P2X7R inhibitors decreased BrdU uptake by either decreasing proliferation or by promoting cell death. Therefore, we performed FACS analysis using Annexin V and propidium iodide and the apoptotic inducer AT101 as control (Fig. 7f). The data show that neither the high concentration of BzATP nor the inhibitors had cytotoxic effects on INS-1E cells. Therefore, the inhibitory effects of high concentration of BzATP, and P2X7R inhibitors, on BrdU incorporation were most likely due to suppression of cell proliferation.

## Discussion

This study shows that INS-1E and β-cells express the P2X7 receptor, and this receptor regulates cell proliferation and insulin secretion, as well as Ca^2+^ signaling and cell metabolism. The receptor also plays a role in glucose-stimulated ATP release, most likely via regulation of Panx1. Furthermore, we show for the first time that Panx1 is expressed in β-cells and plays an important role. The two proteins, P2X7R and Panx1, have key effects in β-cell functions as we discuss in detail below.

Our data show that INS-1E cells express the P2X7R, most likely the full length isoform A (Fig. 1). Other C-terminal truncated isoforms and one with alternative N-terminus has been detected in rodent cells^57,58^. In human cells, there are ten isoforms of P2X7R that may confer different functions^26,59^. INS-1E cells also express Panx1. Panx1 forms hexamers (pannexons), which are transmembrane channels able to release ATP^13^. Three isoforms have been detected: the full-size Panx1A targeted to the plasma membrane and two novel isoforms Panx1C and Panx1D that could potentially form heteromers with isoform Panx1A and regulate its function, as described by Li and coworkers^53^. Immunolocalization experiments show that Panx1 and P2X7R are expressed in INS-1E cells as well as in rat and mouse islets (Fig. 2). We can state that the two proteins are expressed in INS-1E cells but they seem to distribute differently, i.e. Panx1 is more clearly localized to the plasma membrane while distribution of P2X7R is more punctuate. However, using the available antibodies and snapshots of dead cells, we cannot conclude whether in stimulated living cells there could be potential co-localization or interaction of these two proteins, as for example in neuroblastoma cells^60^, or potential interaction with insulin granules during exocytosis.

Interestingly, expression of P2X7R and Panx1 are upregulated with high glucose within two days (Fig. 1). But already within seconds of exposure to glucose these proteins step into action. Exposure to glucose induces a fast (<20 s) and dose-dependent increase in ATP release from INS-1E cells as revealed by on-line ATP measurements (Fig. 3). Part of the release is due to exocytosis of insulin-containing granules, where ATP is accumulated by VNUT. Our evidence supporting this is that the ATP release was partly and significantly abolished by pre-incubation with glyoxylate, a putative VNUT inhibitor, and bafilomycin A. Vesicular ATP release is in agreement with other studies performed on various β-cell preparations^6–9^, and also on exocrine enzyme-secreting pancreatic acini^61^. However, most importantly, our experiments reveal that there is another pathway for ATP release that depends on the P2X7R and Panx1. Panx1 has been described as one of the important proteins responsible for ATP release in other cell types and it can be regulated by various P2 receptors, including the P2X7R either via direct protein-protein interaction or by increase in intracellular calcium^12,13,36,53^. As discussed below, we propose that this is the positive feedback autocrine signal amplifying glucose-induced insulin release^62^.

**Figure 8:**
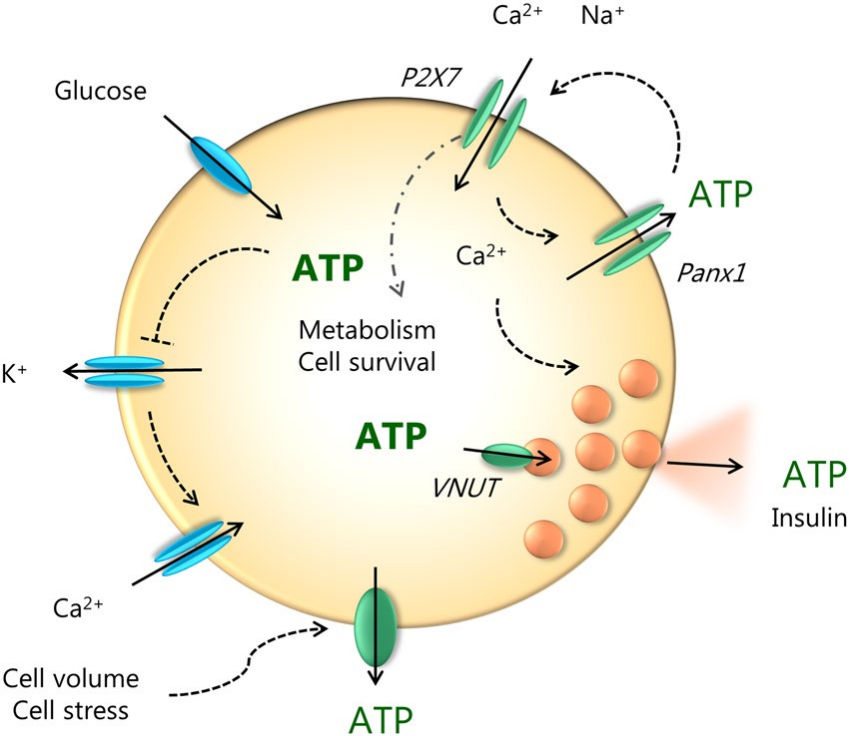
Cell model for autocrine purinergic signaling in β-cells. Glucose enters the cell and is metabolized to ATP. On left side of the cell (in blue) we show the well-established events: closure of ATP-sensitive K^+^ channels leading to cell membrane depolarization, opening of voltage-sensitive Ca^2+^ channels and influx of Ca^2+^ that triggers exocytosis of insulin-containing granules. Granules also contain ATP which is accumulated by VNUT. Our study on INS-1E cells shows (in green) that ATP is also released via pannexin-1 (Panx1) and the process is regulated by the P2X7R. Extracellular ATP binds to the receptor and causes further influx of Ca^2+^ and potentiation of insulin secretion. The resulting amplification of the Ca^2+^ signal promotes additional exocytosis of secretory granules. The P2X7 receptor also regulates β-cell proliferation/survival. ATP can also be released by cell volume/stress, but since these have no effect on cell proliferation, one or more of the steps in the glucose uptake – metabolism – Panx1 - P2X7R – chain are missing.

In many cells, mechanical stress (shear stress) or cell volume changes can also cause significant ATP release^11,63–65^. This is also the case in INS-1E cells where shear stress, cell swelling and cell shrinkage induced ATP release (Fig. 3 and Supplementary Fig. 1). In particular, mannitol in equimolar concentrations as glucose caused cell shrinkage and induced ATP release which was not sensitive to P2X7R, Panx1 or VNUT inhibitors and thus utilized different release pathway to that of glucose-stimulated release. Glucose load had minimal effect on cell volume (Fig. 3d), because after it´s uptake it is rapidly metabolized as shown by the increased intracellular ATP content after stimulation with 16.7 mM glucose (Fig. 3g). In fact, it seems that glucose metabolism is, at least in part, required for ATP release, as a non-metabolizable analogue of glucose 3-OMG and the metabolic uncoupler FCCP significantly reduced both intracellular ATP and ATP release process (Fig. 3f,g). There are a number of studies that have been trying to resolve dynamics in β-cells metabolism, intracellular ATP/ADP levels, calcium signals and exocytosis (see below). Studies using fluorescence reporters like Perceval or luciferase show that there are sub-plasma membrane oscillations in ATP concentrations^66–69^. Origin of these oscillations in β-cells is still unresolved, but one proposal is that it is due to oscillatory glycolysis^66^. Using fluorescent ATP sensors on mouse pancreatic cells, Tanaka and colleagues^70^ showed that glucose induces an increase in cytosolic and mitochondrial ATP (takes about 50–100 s) before onset of calcium oscillations. Interestingly, another study which used similar ATeam sensors targeted to mitochondria of INS-1 832/13 cells shows that there was a small dip in mitochondrial ATP (in about 5–10 sec) before an increase^71^. The authors propose that the dip is due to increased cytoplasmic demand associated with elevated calcium. Our study indicates that this dip could be due to ATP release. In any case, these intracellular events are very fast, and in agreement with the fast transmembrane efflux of ATP we detect. Furthermore, our results show that in INS-1E cells the total intracellular ATP content (metabolism) correlates with ATP release and this in turn has long-term effects on cell metabolism and cell proliferation, and that purinergic signaling is involved in all steps (Fig. 8).

The P2X7R is a ligand-gated ion channel that allows influx of calcium. Accordingly, in INS-1E cells the P2X7R agonist, BzATP, induced synchronized Ca^2+^ transients even at non-stimulating concentrations of glucose, as well as at high glucose concentrations (Fig. 5). This BzATP effect on Ca^2+^ transients was abolished by the P2X7R inhibitor AZ10606120. Most importantly, AZ10606120 and apyrase inhibited Ca^2+^ oscillations induced by high-glucose (Fig. 6) indicating that local ATP release and purinergic system contributes to autocrine signaling. However, we cannot explain how with longer incubations times AZ10606120 causes elevated Ca^2+^ levels. One explanation could be that reduced intracellular ATP (Fig. 3g) could decrease function of the Na^+^/K^+^-ATPase, Ca^2+^-ATPases and Na^+^/Ca^2+^ exchanger, which would lead to imbalance between Ca^2+^ influx and efflux and thus increased and dysregulated cellular Ca^2+^ levels.

Notably, stimulation of the P2X7R with BzATP in INS-1E resulted in insulin secretion, both at low, non-stimulating glucose concentrations, and at high glucose concentrations, where it potentiated glucose effects (Fig. 4). In isolated rat islets, BzATP had similar potentiating effects at low glucose concentrations. Perhaps surprisingly, AZ10606120 pre-treatment did not inhibit high glucose-stimulated insulin release in INS-1E cells, while there was a tendency of inhibition in isolated islets, though not significant. One explanation could be that pretreatment with this blocker also elevates the basal intracellular Ca^2+^ (Fig. 6c), which could maintain overall insulin secretion. Perhaps better time resolution of phasic insulin secretion could contribute to further understanding the role of the P2X7R. Another explanation could be that ATP/BzATP stimulate other P2 receptors that contribute to insulin secretion. Jacques-Silva *et al*.^18^ show that on human pancreatic β-cells apyrase and apyrase inhibitor had negative and positive effects on insulin secretion, respectively, and they proposed that in these cells P2X3 receptors were important in autocrine regulation of insulin secretion.

In addition to the short-term effects on calcium, ATP and insulin release, we show that P2X7R has a role in cell proliferation. Low concentrations of ATP (10–100 μM) significantly stimulated proliferation of INS-1E cells in 5.5 mM glucose. Since cell proliferation was also sensitive to the two P2X7R inhibitors, especially in high glucose medium, it supports the idea that the P2X7R has trophic effects (Fig. 7). This is in agreement with increased expression of P2X7R in high-glucose medium (Fig. 1). In contrast to the P2X7R, Panx1 does not seem to be involved in cell proliferation, although expression of both isoforms Panx1A and Panx1D increased with chronic high-glucose load. While the P2X7R was originally described as a death receptor causing pore-formation and apoptosis/necrosis^41,59^, several recent studies show that the receptor can increase cell proliferation^40,72–75^. Whether this depends on cell type, particular isoform expression, sub-maximal stimulations or a combination of these is not yet clear. Many earlier studies, in particular on immune-reactive cells, show that overstimulation of the receptor (pro-longed exposure or high ATP concentrations) have been associated with cell death^59,76^. However, in INS-1E cells even 1 mM BzATP does not seem to kill the cells, it just decreases their proliferation in both low and high glucose concentrations (Fig. 7).

Taken together, this study shows that the P2X7R is expressed in INS-1E cells (and mouse and rat islets) and affects important cell functions such as ATP metabolism and release, Ca^2+^ oscillations, insulin secretion and cell proliferation. Therefore, one will need to consider function of this receptor in β-cells in normal physiological responses as well as in pancreas pathologies, e.g. diabetes, as P2X7R in immune cells is important in inflammatory processes^25,27,41^. Additionally, the effect of P2X7R SNPs in glucose homeostasis should be considered. It is not yet clear whether the P2X7R signatures will be the same in human and rodent β-cells, as different isoforms may be directing β-cell behavior. In addition, our study reveals expression and function of Panx1 as an additional ATP release mechanism to exocytosis of insulin granules. Panx1 could be regulated by the P2X7R, e.g. via calcium signaling, and calcium influx through P2X7Rs could provide additional nudge to exocytosis of insulin granules (Fig. 7). This feedforward mechanism would potentiate insulin release and may be the mechanism that has been predicted by other studies. Lastly, the bi-phasic effect of P2X7R on proliferation/survival of INS-1E cells is quite remarkable and indicates that similar regulatory switches could exist in other β-cells types. It should be explored whether these would contribute to positive or negative functional outcome in β-cells originating from different species and native cells versus cultured cells. Certainly, above elements of purinergic signaling constitute novel mechanisms for regulation of β-cell mass and enhancement of insulin release and thus could be important targets for diabetes therapy.

## Methods

### Cell culture

The rat pancreatic β-cell line INS-1E (AddexBio, San Diego, CA) passage 30–70 was cultured in RPMI-1640 media supplemented with 10% fetal bovine serum (FBS), 1% penicillin/streptomycin, 10 mM HEPES, 1 mM sodium pyruvate and 50 μM 2-mercaptoethanol. Cells were cultured in a humidified atmosphere at 37 °C with 5% CO_2_ in air.

### Animals

Male Sprague-Dawley rats (100–150 g) and C57BL/6J mice (20–30 g) were obtained from Taconic Biosciences (Ejby, Denmark). The animals had free access to food and water and were killed by cervical dislocation; rats were stunned by hard blow on the head prior to cervical dislocation. All experiments were performed on euthanized animals. Animals were handled according to the guidelines in accordance with EU directive 2010/63/EU on protection of animals used for scientific purposes and experimental protocols were approved by the Danish Animal Experiments Inspectorate (license nr. 2011/561-56).

### ATP release

Extracellular ATP was monitored using luciferin/luciferase luminescence reaction in the extracellular medium. INS-1E cells were plated (50,000 cells/well) on 96-well COSTAR white plates and grown in 5.5 or 2.8 mM glucose 24 h before experiment. Cells were washed and allowed to rest in 65 μl of physiological buffer that contained (in mM): 140 NaCl, 1 MgCl_2_, 1.5 CaCl_2_, 0.4 KH_2_PO_4_, 1.6 K_2_HPO_4_, 10 HEPES and glucose was 5.5 or 2.8 mM in control conditions, pH 7.4. After 30 min 25 μl of luciferin/luciferase mix in physiological buffer (BioThema, ATP kit SL 144–041) was added. Cells were left to rest approximately 20 min or until the luminescence dropped below at least 500 relative luminescence units (RLU). After recording the baseline for 120 sec with low glucose buffer (5.5 or 2.8 mM), 10 μl of the stimulant was pipetted gently to avoid mechanical disturbances. Addition of a buffer containing the same concentration of glucose as in the well served as a “mechanical disturbance” control. The stimulants were buffers containing glucose, such that the final concentration of glucose was 3.5, 5.5, 16.7 or 25 mM in the well. In addition, mannitol and the non-metabolizable glucose-analogue 3-O-methylglucose (3-OMG) were tested. In some experiments cells were pre-incubated with one of the following inhibitors: AZ10606120 (10 μM, Tocris), A438079 (10 μM, Tocris), ^10^Panx (100 μM, Tocris) and FCCP (Carbonyl cyanide 4-(trifluoromethoxy)phenylhydrazone, 5 μM). Measurements were performed using plate mode at 20 sec sampling rate with 1 sec interval time at 37 °C in FLUOstar Optima (BMG, Labtech). All measurements were run in triplicates and n indicates the number of independent experiments. For each experimental protocol a standard curve was made with ATP (ATP standard from BioThema) concentrations ranging from 10^−10^ to 10^−5^ M. The effect of all applied stimuli and inhibitors on luciferin/luciferase assay activity was tested independently on standard curves (in agreement with our earlier study^63^ no effects were observed). Cell numbers were determined by cell counting kit-8 (CCK-8) in parallel wells with same cell seeding, and the concentration of extracellular ATP was corrected to 10^6^ cells per 1 ml. The results are presented as the difference between the basal concentration of extracellular ATP (in 2.8 or 5.5 mM glucose) and the peak concentration of ATP after stimulation (ΔATP released).

### Intracellular ATP measurements

INS-1E cells were treated as described above in “ATP release” assay. After 30 min rest in physiological buffer containing 5.5 mM glucose, cells were pre-incubated with FCCP (5 μM), ^10^Panx (100 μM) or AZ10606120 (10 μM) and then stimulated 2 min with control buffer, 16.7 mM glucose or 16.7 mM 3-OMG. Next, incubation buffer was removed, cells were permeabilized with digitonin (50 μM) and the amount of intracellular ATP was assessed using luciferin/luciferase luminescence reaction (BioThema, ATP kit SL 144–041).

### Cell volume measurement

INS-1E cells were cultured 48 h in RPMI-1640 media (5.5 mM glucose) in μ-Dishes for Live Cell Analysis (ibidi, Germany). After 48 h cells were washed and left in physiological buffer (5.5 mM glucose) containing calcein-AM (5 μM, Molecular Probes C-1430). After 10 min incubation at room temperature cells were washed and suspended in physiological buffer and temperature was regulated at 37 °C. The fluorescent calcein signal (488 nm excitation, 500–540 nm emission) was detected using a confocal laser scanning microscope (SP5-X MP, Leica Microsystems). The following solutions were added: physiological control buffer such that the final concentrations were 5.5 mM glucose, 16.7 mM glucose or 16.7 mM mannitol and hypotonic solution (MilliQ water with 1 mM Mg^2+^ and 1.5 mM Ca^2+^ to achieve 50% dilution). The solutions had the following osmolalities, respectively: 292 ± 2; 305 ± 1; 310 ± 1 and 152 ± 2.7 mOsm/kg (n = 3). The following microscope settings were used during the experiments; 63x oil objective (NA 1.4), zoom factor 3x, format 512 × 512, frame rate 3 sec/frame and speed 400 Hz. 2% laser power and gain 80–95 (HyD2 detection) were kept constant during the experiment.

### RNA isolation and RT-PCR

INS-1E cells were cultured to confluence in a petri dish and RNA was extracted using RNeasy Mini Kit (Qiagen) according to the manufacturer’s instructions. Primers used were as follows: *P*2*X7* (354 bp), 5′- GTGCCATTCTGACCAGGGTTGTATAAA-3′ (forward), 5′-GCCACCTCTGTAAAGTTCTCTCCGAT-3′ (reverse)^77^ and *Panx*1 (125 bp), 5′-TAAACCCCAGCTATGGAGCCA-3′ (forward), 5′-GGCGTCAGTAAAATCCCGTTC-3′ (reverse)^78^. 0.5 μg RNA per reaction was used in OneStep RT-PCR Kit (Qiagen) with amplification parameters as follows: one cycle at 50 °C for 30 min and one cycle at 95 °C for 15 min followed by 35 cycles at 94 °C for 1 min, 50 (Panx1) or 59 °C (P2X7R) for 1 min, 72 °C for 1 min, and finally, one cycle at 72 °C for 10 min. All transcripts were run electrophoretically on 1.2% agarose gels.

### Western blot

INS-1E cells cultured in petri dishes were treated with the indicated concentrations of glucose for 24 or 48 h prior to protein extraction. Protein lysate was prepared and samples were reduced by 10 min heating at 98 °C in the presence of 50 mM DTT (dithiothreitol). Protein (25 μg) was loaded on 10% polyacrylamide precast gels (Invitrogen) and transferred to PVDF membranes (Invitrogen) by blotting. Membranes were blocked with 5% skim milk solution in TBS-Tween (0.1%) buffer for 1 h at room temperature and incubated overnight at 4 °C with primary antibody against P2X7R (1:500 rabbit polyclonal, Alomone APR-004), pannexin-1 (1:1000 rabbit polyclonal, Alomone ACC-234) and β-actin (1:1000 mouse monoclonal C4, Santa Cruz Sc-47778). The blots were incubated with appropriate secondary HRP-conjugated antibodies (1:2500) and developed with EZ-ECL (Biological Industries) and visualized on Fusion FX (Vilber Lourmat). Band intensity was calculated using Bio1D software.

### Immunocytochemistry

INS-1E cells were fixed with 4% paraformaldehyde, permeabilized with 0.1% TritonX-100 and 1% BSA and blocked with 2% BSA in PBS for 1 h. Pancreas was excised from sacrificed animals, fixed in 4% paraformaldehyde, embedded in paraffin. Paraffin sections were de-paraffinized, pretreated with citrate buffer and glycine buffer and blocking buffer. Subsequently, cells and pancreas sections were incubated with antibodies against P2X7R (1:50 to 1:500, Alomone APR-004), pannexin-1 (2–4 μg/ml, HPA016930, Atlas antibodies) and insulin (1:6000, Sigma I2018, K36AC10). Then the samples were incubated with the appropriate secondary antibody conjugated to Alexa 488 (green) or Alexa 568 (red). DAPI (1:400, Molecular Probes) was used as nuclear stain. Fluorescence was examined in Leica SP 5X MP confocal laser scanning microscope with 40 × 1.3 NA or 63 × 1.4 NA objectives and images. Images were collected in sequential scans and analyzed in Leica LAS AF software. Region of interest encompassing whole cells were used for determination of Pearson’s correlation coefficients.

### Cell proliferation

INS-1E cells were plated (6000 cells/well) in white, clear bottom 96-well plates (COSTAR) in RPMI-1640 media containing 5.5 mM glucose. After attachment for 24 h the indicated concentrations of ATP, 2′-3′-O-(4-benzoylbenzoyl)-ATP (BzATP), P2X7R inhibitors (10 μM AZ10606120 and 10 μM A438079) or ATP-diphosphohydrolase (10 U/ml Apyrase, Sigma A6132) were added alone or in combination with high glucose in serum-free media. Cell treated with P2X7R inhibitors were pre-incubated with the inhibitors 15 min before stimulation with glucose. The cells were left undisturbed for another 24 h at 37 °C in a humidified atmosphere with 5% CO_2_ before they were treated with the reagents from Cell Proliferation ELISA, BrdU chemiluminescence kit (Roche) according to the manufacturer’s instructions. All measurements were performed in triplicates. Luminescence was read in FLUOstar Optima (BMG, Labtech).

### Cell viability

Cell viability was estimated using flow cytometry and the Alexa Fluor® 488 annexin V/Dead Cell Apoptosis Kit (Life Technologies). INS-1E cells were cultured in 5.5 mM glucose for 24 h before treatment with the indicated combination of glucose and P2X7R agonists BzATP (100 μM or 1 mM) and inhibitors AZ10606120 (10 μM) and A438079 (10 μM). The apoptotic inducer AT101 (5 μM) was used as negative control. At 24 h after stimulation cells were harvested and stained with Annexin V (488/499) and Propidium iodide (535/617) according to the manufactures protocol. A minimum of 20000 cells per sample were analysed with FlowSight imaging flow cytometer (Merck-Millipore) and IDEAS software was then used to calculate the percentage of live, apoptotic and necrotic populations. Electronic compensation was used to eliminate bleed through of fluorescence.

### Insulin secretion from INS-1E cells

INS-1E were plated (200,000 cells per well) in a 24 well plate and left undisturbed for 72 h. Prior to the experiment, cells were kept 2 h in glucose-free culture medium. Cells were washed and pre-incubated 30 min in Krebs-Ringer bicarbonate HEPES buffer (KRBH) of the following composition (in mM): 135 NaCl, 3.6 KCl, 5 NaHCO_3_, 0.5 NaH_2_PO_4_, 0.5 MgCl_2_, 1.5 CaCl_2_, and 10 HEPES, pH 7.4, BSA (0.1%). Insulin samples were collected from the supernatant after static incubation for a 30 min period in 1 ml of KRBH containing the indicated glucose concentrations alone or in the presence of BzATP (10 μM) or AZ10606120 (10 μM). Samples were stored at −20 °C until measurement of insulin. Samples were analyzed using Rat Insulin ELISA Kit (EMD Millipore) according to the manufacturer’s instructions and including all appropriate standards and controls.

### Insulin secretion from rat islets

Islets of Langerhans were isolated from euthanized adult male Sprague Dawley rats by retrograde perfusion of the pancreas^79^ with 0.5 mg/ml collagenase from *Clostridium Histolyticum* (Sigma, St. Louis, MO) in HBSS (Gibco) supplemented with 5 mM HEPES. The excised pancreas was transferred to HBSS containing 5 mM HEPES, 5 mM glycine and 0.165 mg/ml trypsin inhibitor from *Glycine max* (Sigma) and placed in 37 °C water bath until tissue was readily dissociated when shaken by hand. Digestion was terminated by addition of ice-cold HBSS with 0.4% BSA. Tissue was passed through a size 40 sieve and washed 3 times by centrifugation at 200 *g* for 10 s followed by gradient centrifugation in Histopaque®-1077 at 1000 *g* for 25 min. The islets were washed twice and handpicked under a stereomicroscope. Islets where cultured for 18 h in RPMI1640 medium containing 11 mM glucose, 10% FBS and 1% penicillin/streptomycin in a humidified atmosphere at 37 °C with 5% CO_2_ in air. 11–30 islets were transferred to 300 μl KRBH in low glucose for 2 h prior to addition of the indicated glucose concentrations alone or in the presence of BzATP (10 μM) or AZ10606120 (10 μM). Insulin release was taken as the difference between insulin in the supernatant obtained before addition of test compounds and 30 min after addition. Samples were handled as described above.

### Calcium signals

INS-1E cells were plated on WillCo glass bottom dishes and allowed to attach for 2–3 days. Cells were loaded with Fura-2 AM (2–3 μM) for 30 min in physiological buffer, gently washed and mounted in temperature regulated chamber (37 °C) and most experiments were conducted in standing-bath configuration to minimize the mechanically induced ATP release artifacts (see Supplementary Fig. 1a,b). Experimental solutions contained 2.8 or 16.7 mM glucose and cells were stimulated with BzATP and ATP and some dishes were pre-incubated with the P2X7R inhibitor AZ10606120 (10 μM) or apyrase (10 U/ml). Changes in intracellular Ca^2+^ were followed in Nikon Eclipse Ti microscope with 40x NA1.4 objective. Fura-2 – loaded cells were illuminated at λ_ex_ = 340 nm for 80 ms and λ_ex_ = 380 nm for 60 ms at 1 s intervals (integration of 60 ms for each λ and <1 ms for changing λ) using a TILL Polychrome monochromator. Emission was collected at 500 nm by an image-intensifying, charge - coupled device (CCD) camera (Andor X3 897) and digitized by FEI image processing system (Thermo Fischer Scientific). LA Live Acquisition software was used to both control the monochromator and the CCD camera. The intracellular Ca^2+^ was presented as the ratio of Fura-2 fluorescence signals recorded at 340/380 nm.

### Statistics

Data are shown as mean ± standard error of the mean (s.e.m.) and n denotes the number of independent experiments. Sigma Plot 13 was used for the following analyses. Data were tested for normality. Normalized data were analyzed with One-sample t-tests, followed by correction for multiple comparisons with the Holm-Bonferroni method, when more than two different conditions were tested relative to the control. Non-normalized data were tested with Student’s t tests or One-way ANOVA followed by post-hoc Bonferroni tests. Significant differences *p < 0.05 and **p < 0.01 are indicated.

### Data availability statement

The datasets generated during and/or analyzed during the current study are available from the corresponding author on reasonable request.

## Electronic supplementary material

Supplementary Figures 1–3
