# Supplementary material for: The P2X7 receptor and pannexin-1 are involved in glucose-induced autocrine regulation in β-cells
Source: Sci Rep. 2018 Jun 12;8:8926. doi: 10.1038/s41598-018-27281-9 (PMC5997690; doi:10.1038/s41598-018-27281-9)
Supplement: Supplementary file 1 — Supplementary Figures 1–3 [file 41598_2018_27281_MOESM1_ESM.docx]

**The P2X7 receptor and pannexin-1 are involved in glucose-induced autocrine regulation in** β-**cells**

**Marco Tozzi^#^, Anna T. Larsen^#^, Sofie C. Lange, Andrea Giannuzzo, Martin N. Andersen and Ivana Novak**

**^#^**these authors contributed equally to this work

Section for Cell Biology and Physiology, August Krogh Building, Department of Biology, University of Copenhagen, Denmark

*****Correspondence: Ivana Novak, Department of Biology, Section for Cell Biology and Physiology, August Krogh Building, Universitetsparken 13, University of Copenhagen, DK-2100 Copenhagen, Denmark

Tel: +45 353-30275; e-mail: [inovak@bio.ku.dk](mailto:inovak@bio.ku.dk)

**Supplementary Figure 1**


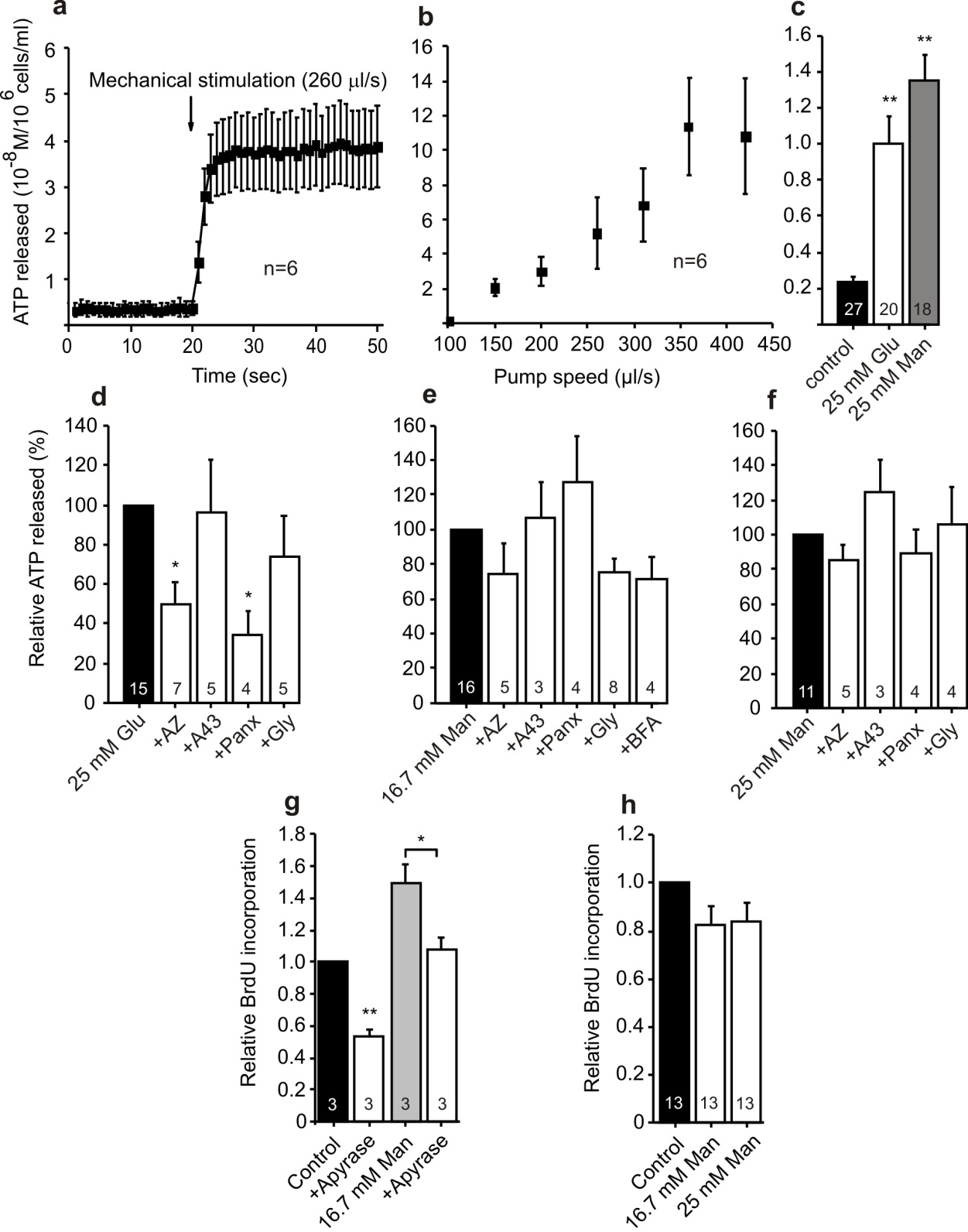


**Effect of various agents on ATP release.** (**a-b**) ATP release caused by mechanical stimulation induced by pump injections (100-360 μl/s) in FLUOSTAR Optima. (**c**) Equimolar concentrations of glucose and mannitol cause similar ATP release. (**d**) Effects of various inhibitors on ATP release induced by 25 mM glucose have similar effects as with 16.7 mM glucose shown in Fig. 3. (**e-f**) Mannitol induced ATP release was not sensitive to P2X7R, Panx1 or vesicular transport inhibitors. **(g)** Apyrase decreased proliferation in cells grown in medium containing 5.5 and 16.7 mM glucose. (**h**) Mannitol had no effect on cell proliferation. Data are shown as means ± s.e.m. of indicated independent experiments and significant differences is indicated p<0.05 (*) and p<0.01 (**).

**Supplementary Figure 2**


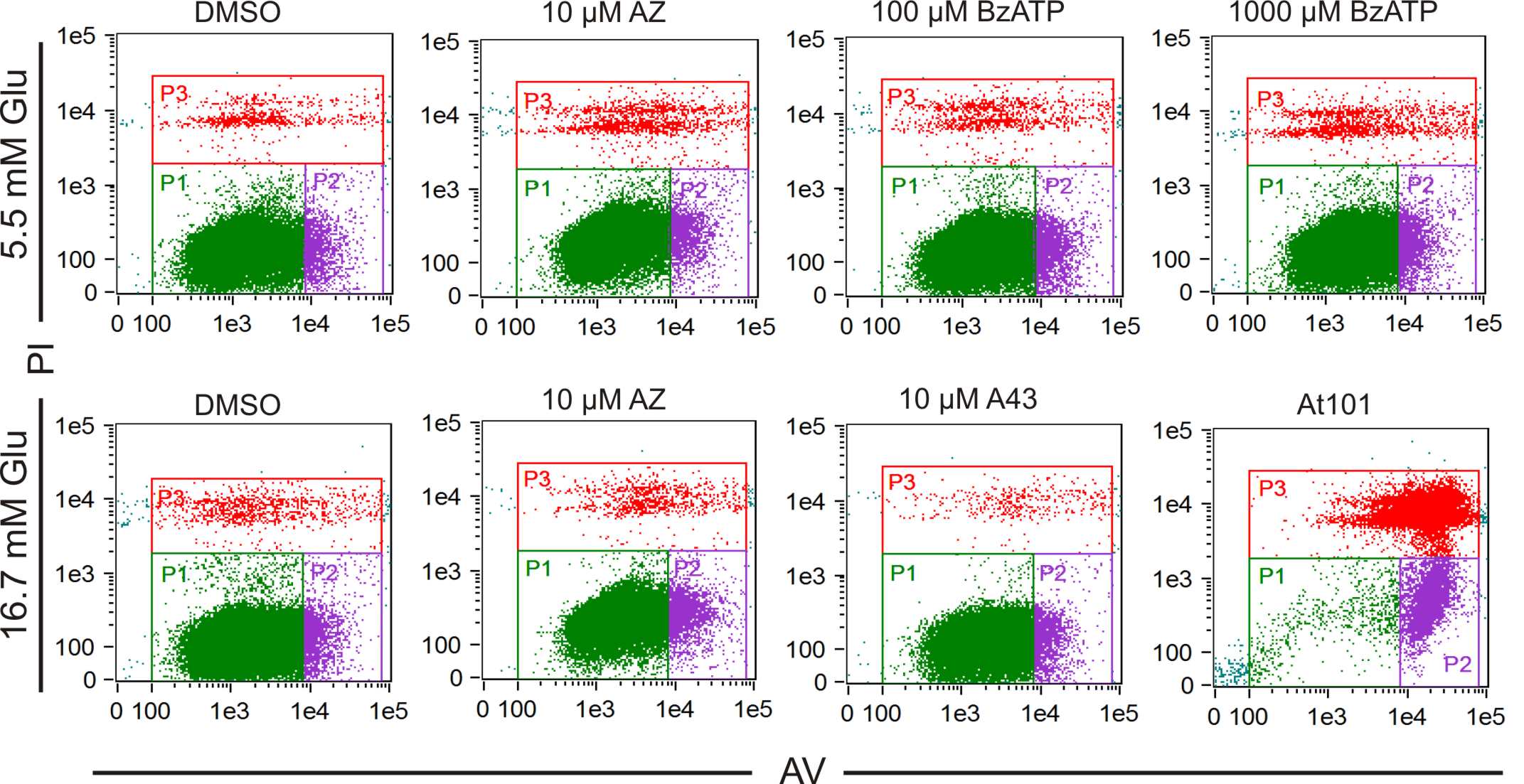


**Representative FACS** plots showing Annexin-V (AV) vs propidium iodide (PI) labelled cells. P1 represents the AV^-^ PI^-^ cell population (live cells); P2 represents the AV^+^ cell population (early apoptotic cells) and the population P3 represents the PI^+^ cells (late apoptotic/necrotic cells). P2X7R was stimulated with BzATP or inhibited with AZ10602120 (10 μM) or A438079 (10 μM). AT101 was used as apoptotic inducer. Samples were analyzed by FlowSight imaging flow cytometer and IDEAS software was then used to calculate the percentage of live, apoptotic and necrotic populations.

**Supplementary Figure 3**


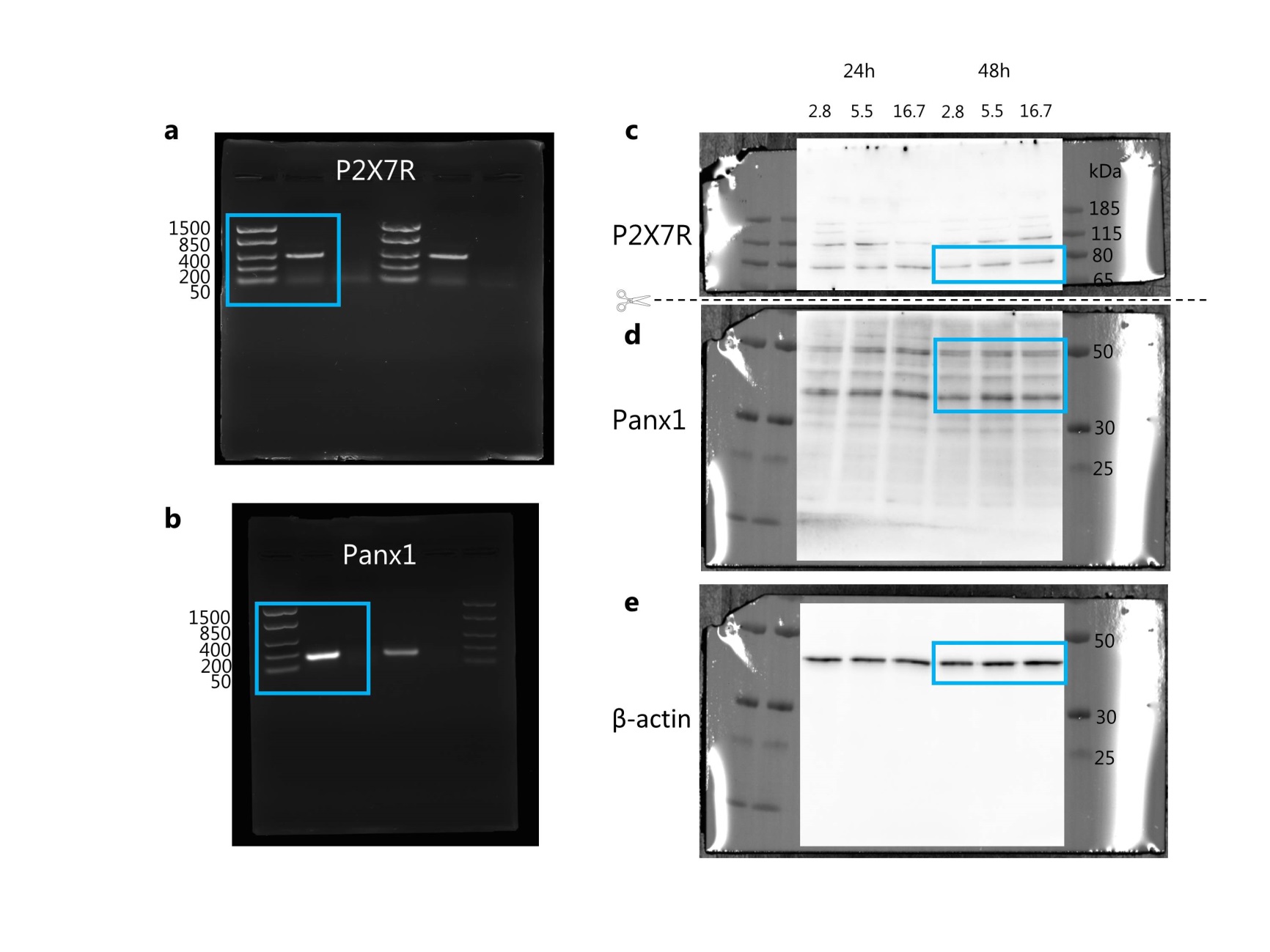


**Expression of P2X7R and Panx1 in INS-1E cells. (a-b)** Representative full-length gels of P2X7 and Panx1 mRNA expression. Highlighted in blue is the part of the picture that has been cropped and showed in figure 1a. The right part of the gel was used for temperature optimization. **(c-d-e)** Representative full-length blot of P2X7R and Panx1 expression in INS-1E cells grown in increasing glucose concentrations. The whole membrane has been cut at 65 kDa (indicated by scissor). The upper part was incubated with P2X7R antibody **(c)** and the lower part with Panx1 antibody **(d)**. The lower part of the membrane was then incubated with β-actin antibody **(e)**. Highlighted in blue is the part of the picture that has been cropped and showed in figure 1b.
